# Current Trends and Future Challenges in Transcatheter Aortic Valve Implantation (TAVI): A Narrative Review

**DOI:** 10.3390/jcm15134850

**Published:** 2026-06-23

**Authors:** Hani Karameh, Prerna Garg, Carla Lucarelli, Mostafa Elguindy, Iqbal Malik, Neil Ruparelia

**Affiliations:** Department of Cardiology, Hammersmith Hospital, Imperial College Healthcare NHS Trust, London W12 0HS, UK; hani.karameh@nhs.net (H.K.);

**Keywords:** TAVI, TAVR, valve durability, coronary access, aortic stenosis, aortic regurgitation, valve in valve TAVI

## Abstract

The advent of transcatheter aortic valve implantation (TAVI) has redefined the treatment of aortic stenosis over the last two decades, evolving from a therapy reserved for patients that were deemed to be of prohibitive surgical risk to the standard of care for a large group of patients presenting with symptomatic disease. With improvements in technology, operator and institutional experience and longer-term outcome data, recent guidelines have supported the broadening of indications to low-risk and asymptomatic patients in addition to other pathologies including the management of failed surgical bioprosthetic valves and aortic regurgitation. The rapid developments in the field have resulted in a rapid expansion of TAVI. The focus has moved from the technical aspects of the procedure itself that are now well established to the lifetime management of patients with aortic stenosis, particularly younger patients with regard to valve durability, planning for a further intervention after TAVI and associated considerations including future coronary access. Beyond aortic stenosis, TAVI technology is also increasingly being utilized for the management of failed surgical bioprostheses, bicuspid valve disease, aortic incompetence and mitral/tricuspid disease and these represent future areas of focus in the field.

## 1. Introduction

Transcatheter aortic valve implantation (TAVI) has rapidly evolved over the last two decades from a treatment reserved for inoperable patients to being central to the management of aortic stenosis across the surgical risk spectrum. It is now the treatment of choice for patients with severe symptomatic aortic stenosis at high and intermediate surgical risk and in patients over the age of 70 [1,2]. Early trials focused on inoperable or high-risk patients [3], demonstrating benefit of TAVI over conservative therapy in this population. Subsequent randomized trials extended these benefits to the intermediate-risk populations [4], and more recently, to low-risk patients, demonstrating comparable clinical outcomes and favourable haemodynamic profiles for TAVI [5,6,7]. This shift has been accompanied by procedural refinements and better care pathways, enabling earlier recovery, a reduced hospital stay and, in selected patients, same-day discharge [8], all with lower complication rates [4,5,6].

Most recently, interest has moved towards the applicability of TAVI technology for the management of younger patients with severe symptomatic aortic stenosis at low surgical risk [9], with some limited recent data supporting this approach [10,11]. Further, the role of treatment of asymptomatic patients has been explored and in certain scenarios has also been associated with clinical benefit [9,12]. Ongoing industry-led trials are also exploring the potential for TAVI in moderate aortic stenosis with symptoms or risk features [13]. The applicability of this technology has also been expanded to the management of patients with bicuspid valve disease, failed bioprosthetic surgical valves and aortic incompetence [14,15]. During this review, we shall summarize the current status of TAVI in relation to its future directions and considerations as this technology expands to different patient groups.

## 2. Methodology

### 2.1. Objective

This article presents a narrative review of current trends and future challenges in TAVI. The aim of this review is to summarize the currently available evidence focusing on clinical outcomes, prosthesis selection, durability, valve-in-valve procedures, and future directions. During the preparation of this manuscript, the authors used Claude AI for language refinement and reference formatting. The authors have reviewed and edited the output and take full responsibility for the content of this publication.

### 2.2. Literature Search

A structured literature search was conducted in PubMed to identify relevant studies evaluating current trends and future challenges in TAVI. SEARCH STRATEGY included combinations of the following keywords: “transcatheter aortic valve replacement,” “TAVR,” “TAVI,” “aortic stenosis,” “self-expanding valve,” “balloon-expandable valve,” “alternative access,” “valve durability,” “valve-in-valve,” and “cerebral embolic protection.” The search was limited to English-language, peer-reviewed articles. Duplicate records were identified by DOI and manually removed. The analysis included clinical trials, prospective and retrospective observational studies, as well as relevant review articles published primarily between 2015 and 2026 to focus on the most recent evidence.

## 3. Current Status

The current 2025 ESC/EACTS (European Society of Cardiology/European Association for Cardiothoracic Surgeons) guidelines [16] provide a Class I recommendation for TAVI in all patients 70 years of age or above with suitable tricuspid anatomy, and a Class IIb recommendation for patients with bicuspid anatomy or severe AR at high surgical risk. The American guidelines remain based on the 2020 American College of Cardiology/American Heart Association (ACC/AHA) recommendations [17]; however, they extend this recommendation to even younger patients, and give a Class I recommendation for shared decision making in patients aged 65–80 years, and in all patients 80 years or older (any surgical risk) after multidisciplinary Heart Team discussion.

Preprocedural evaluation includes a detailed clinical assessment to define the patient profile, symptom burden, and expected benefit of intervention. Baseline electrocardiography (ECG) is essential to identify pre-existing conduction abnormalities and to risk stratify for the potential need for periprocedural pacing. Diagnosis is made by transthoracic echocardiography (TTE) with comprehensive anatomical and haemodynamic assessment. Historically, transoesophageal echocardiography (TOE) was used for annular sizing and intraprocedural guidance; however, this has largely been replaced by multidetector CT (MDCT) and this forms the cornerstone of the preprocedural planning for annular dimensions, calcium quantification, coronary ostial heights, sinus of Valsalva geometry and vascular access route assessment [18].

In current practice, transfemoral access (TF) is the preferred route in the vast majority of patients [19] due to lower complication rates [20,21,22,23]. With improvements in valve technology and delivery systems and the development of vascular closure devices, in combination with greater experience, an increasing number of patients can be treated via transfemoral access with excellent outcomes [19,23,24]. Since the first procedure, sheath size for TAVI has significantly reduced, allowing for a substantial reduction in the minimum size of the calibre vessel required for TF TAVI. With current valve platforms, the minimum diameter required is 5.0 mm for self-expanding technologies that can be advanced with a sheathless approach (14 Fr) and 5.5 mm for balloon-expandable technologies with expanding sheaths with a hydrophilic coating (14–16 Fr) [25,26,27]. Nevertheless, TF access may be unfeasible in 5% to 10% of patients due to severe vascular disease and anatomical limitations [28,29].

**Figure 1:**
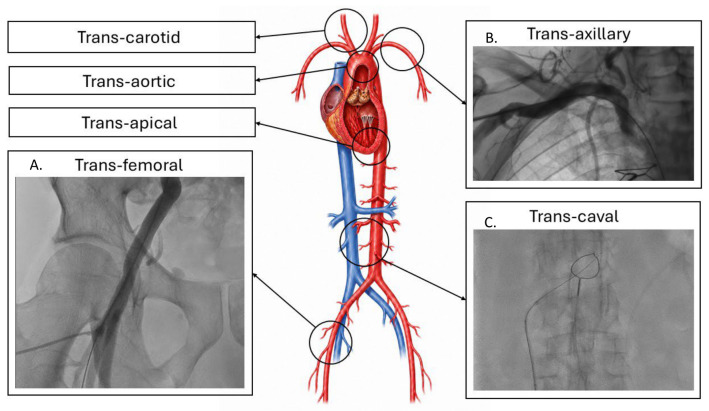
Common vascular access options for TAVI. Panel (**A**): Invasive femoral angiography demonstrating a common femoral artery. Panel (**B**): Invasive angiogram of axillary artery. Panel (**C**): Fluoroscopic image of a coronary wire (Astato 20) in the inferior vena cava that will be used to cross into the aorta using electrosurgery. A goose-neck snare is positioned in the aorta to facilitate transcaval access.

Several alternative routes have been described, including transcarotid, transaxillary, transcaval, transapical and transaortic routes [30,31,32,33]. Furthermore, the shift towards a ‘minimalist’ TAVI approach, including procedures performed under local anesthesia with minimal or no sedation, the use of radial access for secondary access [34], and the use of left ventricular pacing [35], has resulted in less invasive, faster and safer procedures for many patients with significant comorbidities [34]. Figure 1 illustrates the common vascular access option for TAVI.

TAVI device selection has significant consequences for haemodynamic performance, complication profile, and long-term patient management. Understanding the differences between the platforms is clinically important to choose the optimal strategy and device for individual patients. Current devices include balloon-expandable and self-expanding technology and the main characteristics of these are summarized in Table 1. Each of the devices have potential advantages and disadvantages including risks associated with reintervention, long-term durability, valve deterioration, coronary access, aortic regurgitation, paravalvular leak (PVL), patient–prosthesis mismatch (PPM), permanent pacemaker implantation (PPI), and available vascular access and deliverability. The final decision for the valve is made on an individualized basis encompassing lifetime management, vascular access and other comorbidities [36].

Supra-annular valves position the leaflets above the native annular plane, delivering larger effective orifice areas and lower mean gradients—particularly advantageous in small annuli [40]. The SWEDEHEART registry demonstrated that Medtronic Evolut valves achieved the lowest mean gradients in patients with annular diameter ≤ 23 mm (7.97 mmHg vs. 9.02 mmHg for Portico/Navitor and 10.84 mmHg for Acurate; *p* < 0.001) [40]. Intra-annular devices generally offer lower pacemaker rates and less paravalvular leak but higher mean echocardiographic gradients in small annuli [41]. Balloon-expandable valves carry lower pacemaker implantation rates than self-expanding valves (9.9% vs. 17.5%; OR 0.53; 95% CI 0.33–0.86; *p* = 0.009) [42], while differences in moderate-or-greater paravalvular leak (which have been associated with poor outcomes) [43] have largely resolved with current-generation devices due to the development of effective sealing skirts [41].

Long-term outcomes following TAVI continue to evolve as more extended follow-up data become available, particularly across different surgical risk groups and new generation valves. In a recent meta-analysis of TAVI outcomes, in lower-risk patients, TAVI was associated with reduced death or disabling stroke at one year, with no differences beyond one year, while outcomes in higher-risk patients were similar between both groups throughout follow-up [44]. Among low- and intermediate-risk patients, a meta-analysis of 36 studies (seven RCTs) demonstrated that TAVI was associated with lower all-cause mortality than SAVR [45]. In low-risk patients, an updated 2025 meta-analysis demonstrated a significant one-year mortality benefit for TAVI over SAVR, which was attenuated at five years [46]. These data support the view that in higher-risk patients, where the perioperative morbidity of SAVR is considerable, TAVI confers an early clinical advantage. However, in lower-risk patients with longer life expectancy, long-term outcomes between TAVI and SAVR are comparable, at least to 5 years. As discussed in the following section, lifetime management strategies became critical in determining long-term outcomes after TAVI.

### 3.1. Key Challenges

As TAVI expands to younger and lower-risk populations, a number of considerations need to be taken into account and include valve durability, coronary access, the need for pacing, valve anatomy, valve-in-valve strategies for any future procedure, and the possibility of any adjunctive intervention to reduce the risk of stroke.

### 3.2. TAVI Valve Durability and Structural Valve Deterioration (SVD)

Valve durability remains a major concern for lifetime management planning, as it directly influences the possibility of reintervention, particularly in younger, low-risk patients with longer life expectancy. Structural valve deterioration refers to intrinsic, irreversible changes in the prosthetic leaflets, such as calcification, fibrosis, and degeneration, which typically progress with follow-up [47]. In contrast, non-structural valve dysfunction encompasses abnormalities not related to leaflet degeneration, including prosthesis malposition, patient–prosthesis mismatch, and paravalvular regurgitation, which are typically stable over time. Additional mechanisms include leaflet thrombosis, often manifesting as Hypoattenuated Leaflet Thickening (HALT), that is potentially reversible with anticoagulation, and infective endocarditis, which may result in severe valve destruction and require urgent intervention. The development of SVD is multifactorial, involving patient-related factors (younger age, metabolic comorbidities, renal dysfunction), prosthesis-related characteristics (small valve size, high residual gradients, design features), and procedural factors specific to TAVI, such as leaflet injury during crimping, suboptimal expansion, or non-circular deployment. These factors contribute to altered leaflet stress, abnormal flow dynamics, and progressive structural deterioration [48].

The PARTNER 3 trial reported no significant difference in bioprosthetic valve failure at five years (3.3% vs. 3.8%; *p* = 0.62), with severe structural valve degeneration [49] being similarly low in both groups (1.1% TAVI vs. 1.0% SAVR) [5,6]. At ten years, the NOTION trial demonstrated no significant difference in all-cause mortality, stroke, or myocardial infarction (65.5% vs. 65.5%; HR 1.0; 95% CI 0.7–1.3), and the rate of severe SVD was significantly lower with TAVI than SAVR (1.5% vs. 10.0%; HR 0.2; 95% CI 0.04–0.7; *p* = 0.02) [50,51]. Moderate SVD at ten years occurred in 19.4% of TAVI versus 36.0% of SAVR recipients (*p* = 0.0012) [50,52].

There is a note of caution however that the initial studies and recently published 6-year follow up data from the Evolut low-risk trial showed no statistically significant differences in composite endpoint of mortality and disabling stroke between TAVR and SAVR in low-risk patients; however a higher rate of reintervention, largely driven by aortic regurgitation, was noted in TAVI patients, potentially arising from excessive balloon oversizing leading to structural leaflet damage. These findings highlight the importance of extended long-term follow up, strict adherence to manufacturer guidelines and more importantly taking into consideration all potential long-term outcomes in the preprocedural planning [53].

### 3.3. Coronary Access Preservation

Coronary artery disease (CAD) commonly co-exists with severe aortic stenosis, affecting up to 80% of patients undergoing TAVI [54]. The optimal approach to CAD in TAVI remains uncertain. Randomized data suggest no clear benefit of routine preprocedural PCI in stable patients without angina [55], and more recent trial evidence suggested that deferral of PCI was non-inferior to PCI before TAVI for major cardiovascular outcomes after 1-year follow-up [56]. The feasibility of coronary access after TAVI is increasingly relevant, particularly in younger patients with high risk of future coronary intervention, and is influenced by multiple factors including valve design, implantation depth, sinus of Valsalva anatomy, and commissural alignment [56]. Commissure alignment is a surrogate for coronary alignment, since access to the coronaries is the goal. Because the coronary arteries do not always arise from the centre of a sinus, commissure alignment does not guarantee coronary alignment. Intentional commissural alignment at the index procedure reduces coronary ostial overlap from 34.2% to 8.6% (*p* < 0.001) and decreases the need for coronary engagement through struts from 67% to 12% (*p* < 0.001) [57,58], and should be standard practice in eligible patients. Key CT-derived predictors of obstruction include left main height < 10 mm, right coronary height < 12 mm, VTC (virtual valve to coronary) distance ≤ 4 mm, and sinus of Valsalva width < 30 mm [57,59] that may require coronary protection techniques that may make future access even more challenging.

### 3.4. Patient–Prosthesis Mismatch

Patient–prosthesis mismatch (PPM) is defined as an effective orifice area (EOA) indexed to patient’s BSA ≤ 0.85 cm^2^/m^2^ (moderate) or ≤0.65 cm^2^/m^2^ (severe), with failure of the new prosthesis to provide an orifice area adequate for the patient’s haemodynamic demands [60]. Despite its lower incidence with TAVI compared to SAVR due to the absence of a sewing ring (therefore maintaining a larger internal diameter (ID)) which allows deployment of an effectively larger prosthesis within the same anatomical space [61], PPM remains clinically relevant and is associated with reduced left ventricular mass regression, impaired coronary flow reserve, diminished exercise tolerance, and accelerated structural valve deterioration [60,62]. Furthermore, it is independently associated with increased two-year all-cause mortality, heart failure admissions and higher risk of structural valve degeneration [63]. The risk of occurrence is highest in small annuli (<23 mm); patient-level risk factors include high body surface area, female sex, obesity and diabetes, and a bioprosthetic valve rather than a mechanical valve [64]. Supra-annular self-expanding TAVI valves offer greater effective orifice area, better haemodynamic on echocardiographic measurements and a lower incidence of severe PPM compared with balloon-expandable intra-annular designs [61]. A recent publication suggests that invasive haemodynamics are more important and do not show a significant difference between BEV and SEV. Given the substantial impact, prevention where possible is critical and can be achieved with meticulous preprocedural CT-based annular sizing, virtual valve implantation, and anticipated effective orifice area calculation, which are essential tools for identifying patients at risk and informing device selection before the index procedure [61]. Risk factors for PPM are provided in Table 2.

### 3.5. Permanent Pacemaker Implantation

New permanent pacemaker implantation (PPI) remains one of the most common complications after TAVI, with a pooled rate of 18.9% across contemporary series [66]. In recent clinical trials in low-surgical-risk patients, outcomes were favourable for TAVI compared to SAVR; however an excess rate of PPI with supra-annular self-expanding TAVI was noted at 17.4% compared to 6.6% with balloon-expandable TAVI, both of which were higher than SAVR [5,7]. This complication carries an adverse prognostic consequence and is associated with increased one-year mortality (adjusted HR 1.49; 95% CI 1.13–1.97; *p* = 0.005) [67]. Predictors of PPI include pre-existing right bundle branch block, (OR 2.48–4.15), self-expanding valve use (OR 1.93–2.99), deep implantation depth (OR 1.18 per mm) [66], membranous septum (MS) length, male sex, presence of mitral annular calcification and implant depth > MS length [67]. Procedural refinements have been developed to mitigate the risk of PPI with self-expanding valves, such as valve implantation in the two-cusp overlap view. This implantation view isolates the non-coronary cusp and overlaps the right and left coronary cusps to achieve a more precise and higher valve implantation depth in the region where the Bundle of His pierces the membranous septum and hence is more prone to mechanical injury during TAVI [68]. Novel technology continues to evolve to aid in further reducing this risk: the FEOPS HEARTguide, an AI-based software that uses patient-specific CT images to predict risk of conduction abnormalities, shows better predictive value for new-onset conduction disturbances [69], making optimal depth a critical technical target at every procedure.

### 3.6. Bicuspid Aortic Valve

Bicuspid aortic valve (BAV) is the most common congenital heart disease, with a prevalence of 0.5% to 0.8% [70]; recent data from the Society of Thoracic Surgeons Adult Cardiac Surgery Database showed the relative prevalence of BAV in up to 50% of patients undergoing SAVR for severe AS [71]. Patients with BAV are younger; have longer life expectancy; and frequently have complex anatomy with asymmetric annuli, heavy leaflet or raphe calcification, eccentric calcium distribution, eccentric sinuses, and associated aortopathy. Anatomical complexity has historically been classified in view of the number of raphes identified according to the Sievers classification: type 0—true bicuspid, no raphe, and two sinuses; type 1—one raphe; and type 2—two raphes. These features challenge conventional CT-based sizing and procedural planning [65] and increase the risks of paravalvular leak, annular injury, coronary obstruction, pacemaker implantation, incomplete valve expansion, and suboptimal circularity compared with tricuspid aortic valves. [72,73]. Specific sizing methods have been developed for bicuspid anatomies, proposing the true sealing point above the annular level, such as the LIRA (Level of Implantation at the Raphe) and the CASPER (Calcium Algorithm Sizing for bicuspid evaluation valve raphe), which are effective in Sievers type 1 and 2 anatomies. The BAVARD method can be applied to all anatomies and studies the geometry of the annulus and the supra-annular space at 4 mm to identify the sealing point in tubular, flared or tapered anatomies [74,75,76]. Heavy, eccentric, or nodular calcification—particularly at the commissures or extending into the left ventricular outflow tract (LVOT)—raises the risks of annular rupture, coronary obstruction, and incomplete expansion across all anatomies [72,77]. An ascending aorta ≥ 45–50 mm at the time of valve surgery warrants consideration of concomitant aortic root replacement, as TAVI alone leaves a dilated aorta untreated and exposed to future dissection or rupture risk [16,78]. Results are still highly dependent on the baseline anatomy, with Sievers type 0 having better long-term results and Sievers type 1 having increased PVL and risk of annular rupture (0.3%) and valve under expansion [79]. However, long-term results of the NOTION 2 trial showed better surgical results for the bicuspid anatomy. Therefore, the 2025 ESC/EACTS guidelines carry a Class IIb recommendation for TAVI in bicuspid anatomy when surgical risk is increased, and a suitably experienced Heart Team is available [16]. Other anatomical challenges include severe tortuosity of the femoral–iliac axes, and tortuosity of the abdominal aorta. These technical difficulties can be overcome with wire escalation techniques or utilizing devices that have better profiles in navigating tortuous anatomies.

### 3.7. Valve-in-Valve Procedures

The management of surgical bioprosthetic valve failure with valve-in-valve (ViV) TAVI is a well-established safe alternative to redo SAVR in high-risk patients with comparable outcomes in lower-risk patients [80,81,82] with comparable 1-year mortality rates for both balloon and self-expanding valves [83]. This growing field has resulted in an increased preference for biological over mechanical surgical valves at the index procedure [84], and thus avoids long-term anticoagulation therapy, risk of bleeding and thrombosis [85].

Risk of coronary obstruction remains significant in unfavourable anatomies in this subset of patients. Factors include bioprosthetic valve design (externally versus internally mounted leaflets) and bioprosthetic valve alignment to native commissures with a short VTC (<3 mm), and in these circumstances, careful procedural planning with device selection and a strategy for coronary protection should be implemented.

In young patients where valve durability is crucial, achieving a larger EOA avoiding PPM due to the ‘Russian doll’ effect is an essential part of the planning. Valve size selection across all TAVI valve platforms can be achieved using the ViV app, and balloon stent fracture can be performed to maximize EOA in the majority of surgical bioprostheses. The rapid expansion of this field has led to the development of new surgical bioprosthetic valves designed for future valve-in-valve options such as the Inspiris Resilia valve (Edwards Lifesciences, Irvine, CA, USA) [86] with longer-term data with regarding their expected efficacy.

**Figure 2:**
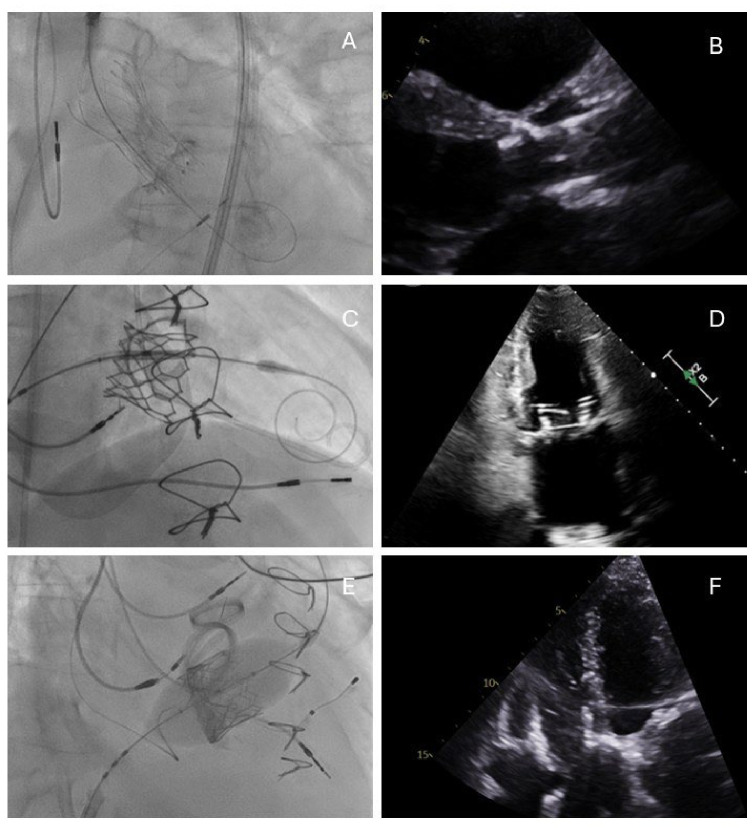
Valve-in-valve (ViV) TAVI, shown on both fluoroscopy and transthoracic echocardiography. Panels (**A**,**B**) show ViV in the aortic position with a self-expanding valve, with the left ventricular delivery wire loop still at the apex. Panels (**C**,**D**) show ViV in the mitral position with a balloon-expandable valve, with a pigtail catheter delivered through the aortic valve to left ventricular apex for invasive haemodynamic assessment. Panels (**E**,**F**) show ViV in the tricuspid position during balloon inflation for valve deployment. Note the mechanical mitral and aortic valve replacements in the background.

The use of ViV-TAVI extends beyond aortic valves to involve mitral and tricuspid valves with high procedural success and acceptable mid-term mortality rates [84]. Transcatheter mitral ViV yields three-year survival of 57.4% with low reintervention rates [87], while tricuspid ViV achieves near-total technical success with durable symptomatic improvement at six years; most survivors remain in NYHA Classes I–II [88]. Redo-TAVI (TAV-in-TAV) is covered below. Figure 2 illustrates valve-in-valve (ViV) TAVI procedures across the aortic, mitral, and tricuspid positions.

### 3.8. Multivalvular Disease (MVD)

Presence of a concomitant valvular disease is frequent in patients with severe AS, which establishes complex disease scenarios requiring careful preprocedural planning and integration of both valve assessments to avoid adverse interactions (e.g., prosthesis–prosthesis interference, coronary occlusion, or conduction disturbances [89]). A recent state-of-the-art review highlights the ongoing challenges in transcatheter mitral valve replacement (TMVI), including patient selection, annular sizing, LVOT obstruction risk assessment, and the need for dedicated devices [90]. The principles of preprocedural CT planning for TMVI are similar to those for TAVR, including assessment of annular dimensions, calcium distribution, landing zone morphology, and neo-LVOT. However, dedicated devices for TMVI are still evolving, and current practice often relies on off-label use of balloon-expandable aortic valves or dedicated mitral valve-in-valve/valve-in-ring systems [90]. LVOT obstruction remains a major concern, but leaflet modification techniques such as LAMPOON have demonstrated an option to overcome these challenges with high short-term technical success and acceptable outcomes, though LVOTO may still occur in up to 10% of cases and longer term outcome data are awaited. Dedicated TMVI devices (e.g., SAPIEN M3) are also emerging [91].

### 3.9. Cerebral Embolic Protection (CEP)

Stroke remains a serious unpredictable complication with an incidence of 3–10% in TAVI cases [92], where almost all patients have radiological evidence of cerebral emboli increasing the risk of cognitive impairment and dementia [92]. With an unclear exact mechanism of cerebral embolism, evidence suggests that approximately half of these events occur in the first 48 h postprocedure, reflecting the direct relation with the procedure itself. CEP devices are designed to attempt to reduce the risk by capturing embolic debris [93]. Current evidence does not support routine CEP device use during TAVI; despite the well-documented embolic burden of the procedure, an updated 2025 meta-analysis of nine RCTs (11,696 patients) found no significant reduction in all-cause mortality, disabling stroke, MACCE, systemic bleeding or neurological outcome. Whilst there may be a role for CEP in specific cases, routine use of currently available devices is not recommended and evaluation of newer devices that provide total cerebral protection are awaited [93].

## 4. Evolving Landscape and Opportunities

### 4.1. Valve Durability

As TAVI expands towards the management of a low-risk and younger patient population, the index procedure is no longer regarded as the definitive treatment but rather the first of a possible sequence of interventions. A lifetime management approach must therefore be adopted and involves careful consideration with regard to device selection, the procedure (implantation technique) and postprocedural management (antithrombotic strategies) to maximize valve longevity and preserve future reintervention options. Each of these steps requires meticulous planning.

Firstly, device selection is dependent upon the collection of anatomical and patient-specific factors, including annular dimensions, coronary anatomy, patient comorbidities, and the geometric requirements of future reintervention.

Secondly, the implantation technique has emerged as an independent determinant of long-term valve durability. Oversized or overexpanded balloon-expandable devices, low deployment depth of self-expanding devices, intra-annular valve position, small annular perimeter, and valve-in-valve deployment are all recognized risk factors for leaflet thrombosis and accelerated structural deterioration [94]. Post-dilation, not uncommonly used to optimize gradients and reduce paravalvular leak (PVL), employs an additional risk of stent frame overexpansion beyond manufacturer specifications and may result in a higher risk of valvular degeneration, with a recent bench study confirming that off-guidance post-dilation of Evolut devices produces measurable leaflet damage proportional to balloon size, with histopathological appearances mirroring those seen in explanted failing valves, reinforcing the fact that preprocedural planning for sizing and deployment is a durability strategy as much as a technical one [95]. Long-term registry data further confirm that both PVL and PPM independently predict adverse outcomes after TAVI, and their prevention remains a clinical need [43,96].

Thirdly, postprocedure management is also important, with antithrombotic management post-TAVI demonstrating an impact upon valve longevity. Subclinical leaflet thrombosis defined as the formation of calcified and non-calcified clots on valve leaflets, detectable on four-dimensional CT as HALT, is observed in up to 20% of TAVI recipients and is associated with reduced leaflet motion, elevated gradients, and potentially accelerated structural deterioration [97,98]. Although anticoagulation suppresses this phenomenon more effectively than antiplatelet therapy, its routine use in patients without an established anticoagulation indication has not led to improved clinical outcomes and carries excess bleeding risk. Current guidelines therefore currently favour single antiplatelet therapy in this group [94,98,99]; however this area remains under active investigation. There are several ongoing trials: ACASA-TAVI (AntiCoagulation vs. AcetylSalicylic Acid after Transcatheter Aortic Valve Implantation) is evaluating anticoagulation versus aspirin in TAVI patients [100]; AVATAR (NCT02735902) is comparing anticoagulation alone against anticoagulation combined with aspirin [101]; ACLO-TAVR (NCT05493657) is directly comparing aspirin and clopidogrel for the prevention of leaflet thrombosis; and the NAPT trial—Non-antithrombotic Therapy After Transcatheter Aortic Valve Implantation—will compare aspirin monotherapy to non-antithrombotic therapy after TAVI [102]. The results of these trials are anticipated to provide a further evidence base needed to refine postprocedural antithrombotic protocols that may further improve valve longevity.

Finally, novel approaches to overcome some of the current limitations are under evaluation. Anti-calcification leaflet treatments (e.g., RESILIA tissue, Edwards Lifesciences) offers a promising opportunity for extending valve longevity. RESILIA tissue, a bovine pericardial material presenting a stable capping of free aldehyde binding sites to resist calcification, has shown reduced structural valve deterioration compared with contemporary bioprostheses in propensity-matched surgical cohorts, with encouraging five-year data from the COMMENCE trial showing superior haemodynamic durability [103,104]. Longer follow-up is required before definitive conclusions can be drawn and current data are limited.

### 4.2. Redo-TAVI and Lifetime Management

Even with strategies to maximize valve longevity, with rapid expansion of TAVI as an alternative to SAVR in low-risk younger patients [49], the life expectancy of this subgroup is likely to exceed the functional lifespan of their bioprostheses [105] and redo-TAVI—transcatheter valve-in-valve implantation within a degenerated index transcatheter prosthesis—will inevitably become an increasingly common clinical challenge [106]. For younger patients, a TAVI-first strategy with planned sequential TAV-in-TAV procedures, reserving surgery for cases where further transcatheter reintervention is not feasible (or where another surgical intervention is concomitantly required), represents an increasingly utilized lifetime management approach [107,108].

**Figure 3:**
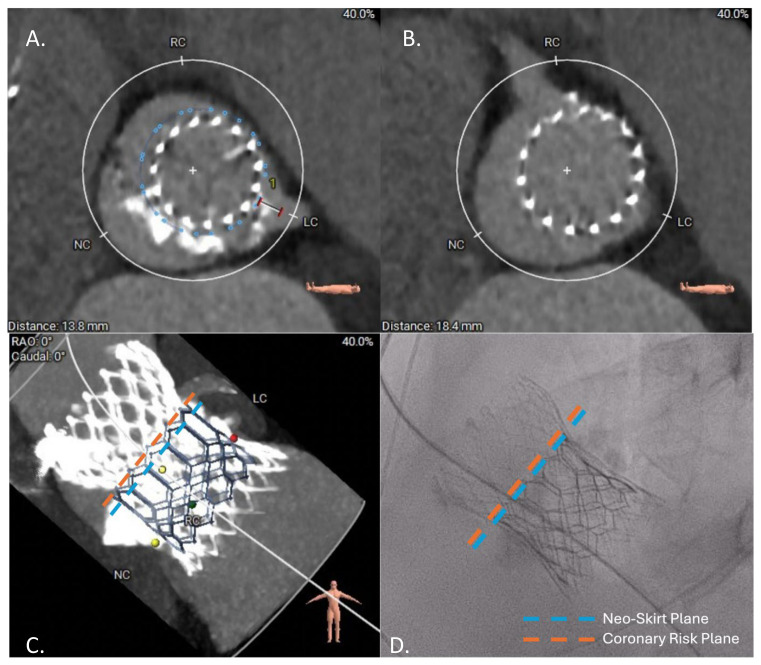
CT-based planning and fluoroscopic guidance for redo transcatheter aortic valve implantation (TAV-in-TAV). Panel (**A**,**B**): CT-derived valve-to-coronary (VTC) distances for the left and right coronary arteries, used to assess coronary obstruction risk. Panel (**C**): Preprocedural CT planning demonstrating a balloon-expandable, short-frame valve within a self-expanding, long-frame valve, with depiction of the coronary risk plane (CRP) and neoskirt plane (NSP). Panel (**D**): Fluoroscopic correlation during the procedure, demonstrating in vivo visualization of the transcatheter valves and the translation of CT-defined CRP and NSP using fluoroscopic markers. LC = Left-coronary cusp; RC = Right-coronary cusp; NC = Non-coronary cusp; Orange-colored dots = Coronary risk plane; Blue-colored dots = Neo-skirt Plane.

As a result, attention has moved toward safe and effective strategies for TAV-in-TAV procedures. This requires a systematic, CT-based approach beginning with detailed characterization of the index valve, including valve type and size, failure mechanism, and commissural and coronary alignment [109]. Considerations in the selection of the second valve and implantation depth include coronary risk, leaflet overhang, and haemodynamics. Careful assessment of coronary risk is needed, by defining the coronary risk plane (CRP) and its relationship to the neoskirt plane (NSP) in order to avoid coronary obstruction and sinus sequestration. When the NSP lies above the CRP, further evaluation of valve-to-aorta distances is essential. Strategies to mitigate risk include lowering the implantation plane, leaflet modification techniques, changing the valve type, or implementing coronary protection strategies. If coronary risk is prohibitive, surgical explant of the TAVI prosthesis may be required [49]. Figure 3 illustrates CT-based planning and fluoroscopic guidance for redo transcatheter aortic valve implantation (TAV-in-TAV).

Emerging data on TAV-in-TAV outcomes are promising. The RE-TAVI registry, the largest prospective data source of repeat TAVI with BEV, reported a 30-day mortality of 3.4% and one-year all-cause mortality of 15.5% in 464 patients, with high procedural success and acceptable haemodynamic outcomes, though coronary obstruction and conduction abnormalities remained the principal safety concerns [110]. The transcatheter approach to degenerated bioprostheses has now been extended beyond the aortic position, with valve-in-valve procedures for failed mitral and tricuspid bioprostheses increasingly performed at experienced centres with acceptable procedural success and mid-term outcomes [111]. Ongoing trials aim at providing more robust data to evaluate the treatment of valve deterioration by redo-TAVI in a global prospective study that will likely provide data required to define best practices for reintervention [112].

### 4.3. Leaflet Modification

**Figure 4:**
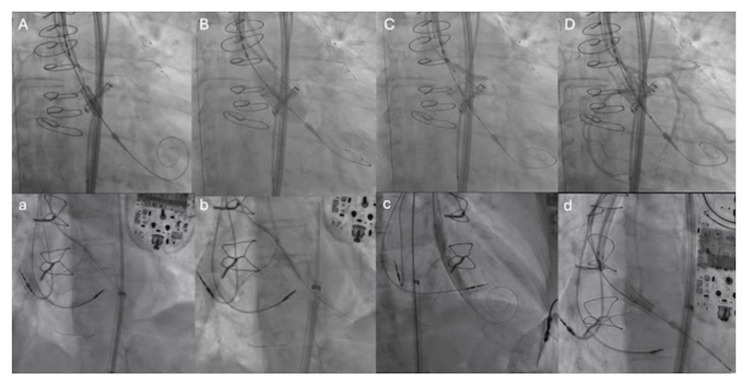
Contemporary strategies of coronary protection in ViV TAVI: bilateral chimney stenting (**A**–**D**) and UNICORN (**a**–**d**). (**A**). RCA and LMS protection: RCA: JR4—BMW RCA—Guideliner 3.5 × 23 mm stent at ostial RCA, angulated with aorta. LMS: JL3.5, Sion blue—Guideliner and 4.0 × 23 mm stent—parked in LMS. (**B**). Stents in position at valve deployment, guide catheters pulled back, 20 mm BE TAVI deployed. (**C**). Stents deployed to LMS and RCA. (**D**). Final result, with excellent coronary flow bilaterally. (**a**). LAO cranial view 8 Fr Amplatz left catheter to image LCA sinus. Finecross and electrified Astato wire to cross left leaflet. (**b**). Exchanged for BMW wire and predilatation with 4.5 mm coronary balloon at 14 atm. (**c**). Further dilatation with 10 mm Mustang balloon to 16 atm. (**d**). Successfully deployed 20 mm BE valve.

Preventive strategies for coronary obstruction have expanded considerably in recent years beyond coronary protection and bailout stenting, and whilst feasible are associated with complications including stent re-stenosis, thrombosis and very challenging (if not impossible) re-access [113]. With the advent of electrosurgery, bioprosthetic or native aortic scallop intentional laceration to prevent iatrogenic coronary artery obstruction (BASILICA) [114] has been demonstrated to be a very effective and safe method to reduce the risk of coronary obstruction in high-risk anatomy. More recently, there have been further alterations of the techniques, including Undermining Iatrogenic Coronary Obstruction with Radiofrequency Needle (UNICORN) and leaflet laceration with balloon-mediated annihilation to prevent coronary obstruction with radiofrequency needle (LLAMACORN), [115,116]. Most recently, dedicated devices including ShortCut [117] and the Transmural Electrosurgery LeafLet Traversal And Laceration Equipment (TELLTALE) [118] system have been used to further simplify the process of leaflet modification. The TELLTALE trial reported a primary efficacy endpoint of 100% (95% CI 95–100%) with 0% 30-day mortality [118]. Each of these options appear to be safe, reproducible and effective in the short-term, and longer-term data are awaited to determine if this can be regarded as a safe ‘first-line’ approach. Figure 4 illustrates contemporary strategies of coronary protection in ViV TAVI.

Electrophysiology techniques have been further expanded to prevent LVOT obstruction in unfavourable anatomies (neoLVOT < 200 mm^2^) caused by the anterior mitral leaflet during procedures in the mitral position. Analagous to BASILICA, the Laceration of the Anterior Mitral Leaflet to Prevent Outflow Obstruction (LAMPOON) uses electrosurgery to produce a longitudinal laceration of the anterior mitral leaflet. The more recent Balloon-Assisted Translocation of the Mitral Anterior Leaflet (BATMAN) procedure is a simplified alternative which, after perforation of the anterior mitral leaflet, uses sequential balloon inflations to translocate the leaflet from its original position during TMVR with an Edwards valve [119,120].

### 4.4. Aortic Regurgitation

The off-label use of TAVI technology for the management of aortic regurgitation has occurred for many years [121]. Procedures however have been complicated with high rates of device embolization, pacing and failure [122]. Recently, dedicated devices for native aortic regurgitation have been developed and represent a distinct emerging category. The JenaValve Trilogy system incorporates locator technology that enables anchoring without annular calcium, and the ALIGN-AR pivotal trial of 700 high-risk patients demonstrates a technical success rate of 99.1% and 30-day all-cause mortality of 1.6% [15]. Longer-term outcomes including valve durability for this indication are awaited to determine if TAVI can be considered a first-line treatment for these patients.

### 4.5. Next-Generation Valve Technologies

To overcome some of the limitations of current-generation devices, specifically durability, there is a growing interest in the development of non-tissue percutaneous devices. Polymeric heart valves (PHVs) aim to combine the haemodynamic performance of current platforms in addition to increased durability and reduced thrombogenicity, calcification and structural fatigue. This represents a compelling long-term durability solution, particularly for younger patients. Preclinical testing with polyurethane-based nanocomposite valves has been incorporated into devices including the Triskele and Polynova valves, the latter exceeding 400 million cycles in accelerated wear testing, showing resistance to calcification and less thrombogenicity [123,124,125]. The Foldax Tria valve remains the only PHV surgical implant in humans to date (14 patients), with preserved haemodynamic gradients at one year, while its transcatheter valve has completed bovine preclinical studies ahead of planned first-in-human trials [126,127]. Longer-term data with regard to both safety and efficacy are currently lacking [124]; see Table 3.

## 5. Areas of Future Focus

Many of the initial technical challenges of TAVI have been addressed and the focus of future research will centre upon broadened indications of the technology, patient groups that would benefit from intervention (and those that would not) and longer-term planning. Emerging 3D holographic mixed-reality platforms which allow operators to interact with patient-specific cardiac issues may be helpful in preprocedural planning in challenging complex anatomies such as BiV, ViV procedures, or patients at risk for coronary obstruction. In a recent study, holographic mixed-reality for TAVI planning demonstrated good feasibility and accuracy compared with standard MDCT measurements, and operators reported improved spatial understanding of anatomically challenging cases [128].

The EARLY TAVR trial established that early intervention in asymptomatic severe aortic stenosis was superior to clinical surveillance for the primary composite endpoint (HR 0.50; 95% CI 0.35–0.72; *p* < 0.001), providing the first randomized evidence to support treatment before symptom onset and prompting regulatory approval in this indication [124]. Whether this approach extends to moderate aortic stenosis to prevent myocardial damage and improve clinical outcomes is currently under investigation [129,130]; the results of these trials may alter the approach of AS, shifting the care from a valve severity- and symptom-guided approach to a myocardial cellular-level assessment. The 2025 ESC/EACTS guidelines have lowered the TAVI age threshold from 75 to 70 years (Class I for tricuspid anatomy with suitable transfemoral access) [16], a change that will substantially expand the cohort of patients for whom TAVI is the guideline-preferred option and who will require decades of structured follow-up.

Same-day discharge, associated with lower 30-day mortality (OR 0.104), lower readmission (OR 0.194), and lower new pacemaker requirement (OR 0.167) compared with next-day discharge in a meta-analysis of 3519 patients [8], is increasingly explored in appropriately selected patients, and longer-term safety data are awaited to determine if this becomes the standard of care for the significant group of patients undergoing transcatheter valve therapy.

## 6. Limitations

This review has certain limitations. We did not perform a meta-analysis, and the search for articles was not systematic, so some relevant studies may have been missed. Selection of references was based on the authors’ knowledge, which could introduce bias. Finally, because TAVI technology is evolving rapidly, new evidence may have appeared.

## 7. Conclusions

There has been remarkable progression in the TAVI field from the first description nearly twenty years ago to the current day where TAVI has moved from being reserved as a ‘no option’ procedure for patients presenting with severe symptomatic aortic stenosis deemed to be of prohibitive surgical risk to the standard of care for patients with severe AS across all risk categories. Evidence supporting this evolution is now substantial, and as TAVI expands into younger populations, the current practice of care will now appropriately focus on lifetime management.

Optimal outcomes require a multidisciplinary Heart Team; individualized, patient-centred care; preprocedural planning; appropriate device selection; and adopting optimal implantation techniques.

As the field continues to evolve, future research will focus upon the management of patients with asymptomatic disease, earlier intervention with patients with moderate disease, dedicated devices for native aortic regurgitation, coronary access, leaflet modification techniques, and same-day discharge protocols whilst carefully defining longer-term durability data that will ultimately determine the optimal management of patients with valve disease.

## Figures and Tables

**Table 1:** Contemporary transcatheter heart valve platforms—key design features and performance attributes.

Device (Manufacturer)	Type	Position	Material	Sizes	Annulus Range (mm)	Stent Frame Height (mm)	Recapturable/Repositionable
SAPIEN 3/Ultra (Edwards) [37]	Balloon-expandable	Intra-annular	Bovine pericardium (cobalt–chromium frame)	20 mm, 23 mm, 26 mm and 29 mm	18–28	15.5–22.5	No
Evolut R/Pro/FX/FX + (Medtronic) [37]	Self-expanding	Supra-annular	Porcine pericardium (nitinol)	23 mm, 26 mm, 29 mm, and 34 mm	18–30	45–46	Yes (<80% deployed)
Navitor (Abbott) [37]	Self-expanding	Intra-annular	Bovine pericardium (nitinol)	23, 25, 27, 29, and 35 mm	23–29 (35 mm Titan)	47–48	Yes (≤80% deployed)
Portico (Abbott) [37]	Self-expanding	Intra-annular	Bovine pericardium (nitinol)	23 mm, 25 mm, 27 mm, and 29 mm	18–25	47–48	Yes (≤80% deployed)
JenaValve (JenaValve) [37]	Self-expanding	Intra-annular	Porcine aortic root (nitinol)	23 mm, 25 mm, and 27 mm	21–27	------	No
Allegra (NVT AG) [38]	Self-expanding	Supra-annular	Bovine pericardium (nitinol)	23 mm, 27 mm, and 31 mm	-------	------	No (early valve function)
Myval (Meril Life Sciences) [39]	Balloon-expandable	Intra-annular	Bovine pericardium (cobalt–nickel alloy)	Conventional (20, 23, 26, 29 mm), Intermediate (21.5, 24.5, 27.5 mm), Extra-large (30.5, 32 mm)	20–32	------	No
Hydra (SMT)	Self-expanding	Supra-annular	Bovine pericardium (nitinol)	22 mm, 26 mm, and 30 mm	18–27	------	Yes

**Table 2:** Risk factors for patient–prosthesis mismatch (PPM) after TAVI.

Risk Factor	Description/Mechanism
Small aortic annulus	Annular perimeter < 72 mm or area < 400 mm^2^ [40,61]
Small body surface area (BSA)	Lower BSA increases indexed mismatch severity (same EOA yields higher PPM grade) [64]
Female sex	Smaller annular dimensions and lower BSA; accounts for 80–90% of small-annulus patients [61,64]
Small prosthesis size	≤23 mm (balloon-expandable) or ≤26 mm (self-expanding) directly limit effective orifice area [64,65]
Intra-annular valve design	Supra-annular designs achieve larger indexed EOA for the same annular size [61]
Heavy annular/leaflet calcification	Prevents full frame expansion, reducing achieved EOA [40,63]
Bicuspid aortic valve	Asymmetric annulus and irregular calcification; may impair valve seating and expansion [40]

BSA = body surface area; EOA = effective orifice area; and PPM: patient–prosthesis mismatch.

**Table 3:** Emerging transcatheter valve platforms.

Device	Company	Type	Indication	Key Innovation	Trial/Status
DurAVR (Anteris)	Anteris/Medtronic	BE	AS	Single-piece moulded biomimetic ADAPT tissue leaflet; near-physiological flow on cardiac MRI; designed for redo-TAVI access	PARADIGM RCT
					Enrolling; 1000 pts; 1:1 vs. approved TAVI
Trilogy (JenaValve)	JenaValve Technology	SE; leaflet-grasping	AR (native)	Cusp-grasping locators anchor to native leaflets without calcification; dual-disease capability (AS + AR); large open cell for coronary access	ALIGN-AR pivotal
					700 pts; 2 yr composite endpoint met; published Lancet 2025 [15]
Myval (Meril)	Meril Life Sciences	BE	AS; ViV; bicuspid; AR	Hybrid hexadecagonal frame; 0.5 mm size increments (12 sizes); designed for versatility across complex anatomies and ViV procedures	MyVal-1/LANDMARK RCT
					LANDMARK non-inferiority vs. SAPIEN 3/Evolut ongoing
Foldax Tria (TAVI version)	Foldax	Polymeric—BE	AS	Synthetic siloxane polyurethane-urea (SiPUU) leaflets; no biological tissue; anti-calcification by design; first PHV tested in humans (surgical version, 14 pts)	Preclinical
					Ovine preclinical complete; first-in-human TAVI trials planned

PHV = polymeric heart valve; ViV = valve-in-valve; AS = aortic stenosis; AR = aortic regurgitation; BE = ballon-expandable; SE = self-expanding.

## Data Availability

The original contributions presented in this study are included in the article. Further inquiries can be directed to the corresponding author.
